# How Antioxidants, Osmoregulation, Genes and Metabolites Regulate the Late Seeding Tolerance of Rapeseeds (*Brassica napus* L.) during Wintering

**DOI:** 10.3390/antiox12111915

**Published:** 2023-10-26

**Authors:** Pengfei Hao, Baogang Lin, Yun Ren, Hao Hu, Weidong Lou, Kaige Yi, Bowen Xue, Lan Huang, Xi Li, Shuijin Hua

**Affiliations:** 1 Institute of Crop and Nuclear Technology Utilization, Zhejiang Academy of Agricultural Sciences, Hangzhou 310021, China; 11816004@zju.edu.cn (P.H.); linbg@mail.zaas.ac.cn (B.L.); kaige_y0113@163.com (K.Y.); tianyuxingcheng@foxmail.com (B.X.); huang975885829@163.com (L.H.); 202200101021@stu.xza.edu.cn (X.L.); 2 Huzhou Agricultural Science and Technology Development Center, Huzhou Academy of Agricultural Sciences, Huzhou 313000, China; yunhuaren@163.com; 3 Institute of Digital Agriculture, Zhejiang Academy of Agricultural Sciences, Hangzhou 310021, China; huh@mail.zaas.ac.cn (H.H.); louwd@zaas.ac.cn (W.L.)

**Keywords:** agronomic characters, multi-omics, oil security, phenylpropanoid biosynthesis, ROS, TCA cycle

## Abstract

Rapeseed seeding dates are largely delayed under the rice–rape rotation system, but how rapeseeds adapt to the delayed environment remains unclear. Here, five seeding dates (20 October, 30 October, 10 November, 20 November and 30 November, T1 to T5) were set and the dynamic differences between two late-seeding-tolerant (LST) and two late-seeding-sensitive (LSS) rapeseed cultivars were investigated in a field experiment. The growth was significantly repressed and the foldchange (LST/LSS) of yield increased from 1.50-T1 to 2.64-T5 with the delay in seeding. Both LST cultivars showed higher plant coverage than the LSS cultivars according to visible/hyperspectral imaging and the vegetation index acquired from an unmanned aerial vehicle. Fluorescence imaging, DAB and NBT staining showed that the LSS cultivars suffered more stress damage than the LST cultivars. Antioxidant enzymes (SOD, POD, CAT, APX) and osmoregulation substances (proline, soluble sugar, soluble protein) were decreased with the delay in seeding, while the LST cultivar levels were higher than those of the LSS cultivars. A comparative analysis of transcriptomes and metabolomes showed that 55 pathways involving 123 differentially expressed genes (DEGs) and 107 differentially accumulated metabolites (DAMs) participated in late seeding tolerance regulation, while 39 pathways involving 60 DEGs and 68 DAMs were related to sensitivity. Levanbiose, α-isopropylmalate, s-ribosyl-L-homocysteine, lauroyl-CoA and argino-succinate were differentially accumulated in both cultivars, while genes including isocitrate dehydrogenase, pyruvate kinase, phosphoenolpyruvate carboxykinase and newgene_7532 were also largely regulated. This study revealed the dynamic regulation mechanisms of rapeseeds on late seeding conditions, which showed considerable potential for the genetic improvement of rapeseed.

## 1. Introduction

Global agriculture production is significantly threatened by changes in the climate and environment, which, without adaptation, are expected to decrease agricultural productivity and raise the risks of crop failure [1,2]. According to existing research, 39% of global crop fields require new varieties to avoid a loss of yield from climate change by the end of the century to ensure food security [3,4]. Asseng et al. (2015) suggested that varieties required more heat units to delay maturity and extend grain development in order to adapt to increased temperatures [5]. According to a case in the Yangtze River Basin (YRB), China, rice–rape rotation is the dominant cropping method; however, farmers have delayed rice harvests to gain a higher yield and grain quality as global warming intensifies, which has led to higher requirements for rapeseed production [6,7]. According to a previous report, as seeding dates are delayed, extreme weather events will occur more frequently, especially when wintering, which will result in widespread reductions in yield and even crop failure [2,8].

Rapeseed (*Brassica napus* L., AACC, 2*n* = 38), as an oil crop grown in winter, provides over 47% of the domestic plant edible oil in China. In addition, as rapeseed does not compete with summer staple crops such as rice for land, it is regarded as the most promising winter rotation crop [9,10]. However, as rice harvest time is delayed, the seeding dates of rapeseed will be postponed to as late as December, while its suitable seeding dates are late September to early October [11]. Meanwhile, the maximum air temperature decreases very quickly from near 30 °C in November to even below 5 °C in December, which greatly inhibits the production of rapeseed. As reported, the delay in planting greatly inhibited the photosynthetic rates of rapeseed and led to a dramatic reduction in pod numbers [12,13]. In addition, the oil content has been reported to have reduced by 0.5–1.5% with a one-week delay [14], seriously limiting the production of rapeseed [15]. Therefore, how rapeseed adapts to delayed seeding environment conditions, especially when wintering, has become a vital area of study for future oil security and sustainability.

It is worth mentioning that, due to global changes and the low planting enthusiasm of farmers, over 100 million acres of winter fallow fields have been generated in the YRB, of which about 50 million acres can be planted with rapeseed, and if they are fully utilized, over 15.17 million tons of rapeseed oil, which will account for more than 7% of the vegetable oil production of the world, can be produced [16]. Therefore, the adaptation of rapeseed to complex environmental conditions can not only help to improve rapeseed yield, but also benefit the utilization of the large area of winter fallow fields, and hence ensure oil security and sustainability.

Among the different adaptation measures in agriculture, variety adaptation has been identified as one of the most effective, and region-specific breeding efforts are needed to achieve an effective adaptation to climate change for sustainability [1,17]. Illustrating the late seeding response mechanisms and breeding tolerance genotypes under late seeding conditions are necessary for food and oil sustainability. Tian et al. (2021) first quantified the extent of winter fallow fields and identified the fallow periods in winter, while assessing the great potential of rapeseed production in the YRB [16]. Luo et al. (2022) provided a more reliable comprehensive index for cold-tolerance evaluation through measurement of the growth parameters during germination and the emergence stage of 436 natural rapeseed populations, based on a pot experiment [10]. In addition, Zhang et al. (2019) evaluated 12 seed-germination- and seedling-emergence-related indices of 132 *Brassica napus* genotypes under normal and low-temperature conditions and screened out three highly low-temperature-tolerant genotypes [18]. For further illustration, Qin et al. (2023) identified the phenotypic damage and transcriptome profiles of two rapeseed inbred lines in the early flowering stage under cold stress through phytotron experiments, and 1396 DEGs were identified [19]. Similarly, Mehmood et al. (2021) used iTRAQ and transcriptomics to identify 48 DEGs and 17 DEPs corresponding to a cold-tolerant rapeseed line and 82 DEGs and 38 DEPs in a cold-sensitive line at the four-leaf stage in a climate chamber [20]. Furthermore, the effects of antioxidant enzymes, sugar metabolism, the CBF-COR pathway and MAPK signaling on resistance to low-temperature conditions were also identified based on light incubator conditions [21,22].

However, most of these studies were conducted in light incubators, phytotron or climate chambers, which cannot entirely reflect the complex field environment conditions, and contrasting appearances will also occur in field conditions. In addition, late seeding stress contains multiple abiotic and biotic factors, such as cold, chilling, raining, drought and so on, not merely cold or chilling. Against this background, we aimed to identify and characterize the late seeding response mechanisms of rapeseeds (*Brassica napus* L.) by comparing two late-seeding-tolerant with two late-seeding-sensitive cultivars at five seeding dates. By evaluating their wintering ability according to the measurement of agronomic characters, including multi-spectral, osmoregulation, ROS and antioxidant, the physiological mechanisms were revealed. Furthermore, the interaction analysis of transcriptome × metabolome was also studied, aiming to disclose the adaptation mechanisms of rapeseeds in the YRB under late-seeding-environment conditions. In our view, this is the first study which comprehensively studied the dynamic changes in physiology, genes and metabolites during wintering under field conditions, which will help to identify the merits of late-seeding-tolerant rapeseed and provide fundamental references for future rapeseed breeding.

## 2. Materials and Methods

### 2.1. Experimental Site, Plant Materials and Experimental Design

#### 2.1.1. Conditions of Experiment Site

The field trials were conducted in Yuhang District (30°9′~30°33′ N, 119°40′~120°8′ E), Zhejiang, China, which has a subtropical monsoon climate with an average annual rainfall of 1150–1550 mm and an average annual temperature of 15.3–16.2 °C. Rice–rapeseed rotation has been practiced for a long time in this area.

#### 2.1.2. Plant Materials

Four rapeseed (*Brassica napus* L.) genotypes were used: V1, Fengyou 737 (control 1, FY737, late-seeding-tolerant (LST) cultivar, which is widely cultivated as a control cultivar in China); V2, Zheyouza 1510 (ZYZ1510, late-seeding-tolerant material to be tested, newly developed by our group); V3, Zheyou 50 (control 2, ZY50, late-seeding-sensitive (LSS) cultivar, nationally the main recommended backbone rapeseed cultivar since 2014); and V4, B11 (late-seeding-sensitive material to be tested, newly developed by our group).

#### 2.1.3. Experimental Design

A split-plot experiment was conducted with 5 seeding times as the main plot (T1, 20 October; T2, 30 October; T3, 10 November; T4, 20 November and T5, 30 November) and 4 rapeseed materials as split plots. There were 3 replicates, and the area of each was 20 m^2^ (2 m × 10 m). Strip sowing was adopted, and 250 thousand plants per hectare remained at the seedling stage.

**Figure 1:**
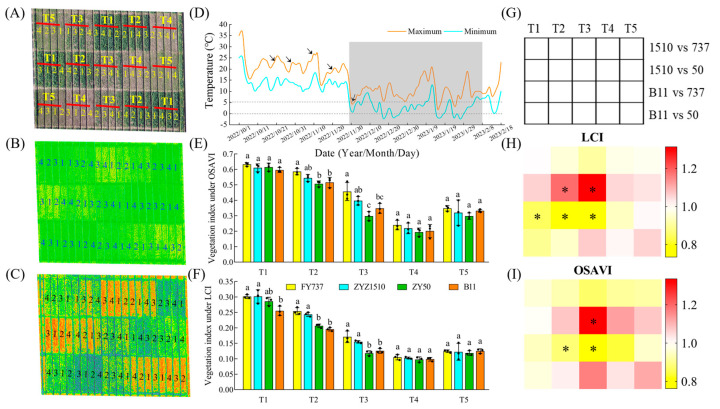
The phenotype of different rapeseed materials under natural cold-stress photographed by unmanned aerial vehicle. (**A**) Field planting model under UAV imaging; (**B**,**C**) aerial hyperspectral image and vegetation index under LCI and OSAVI; (**D**) variation of temperature in Hangzhou Yuhang district from seeding time to the end of cold. arrows represent the five seeding dates. (**E**,**F**) vegetation index obtained from aerial hyperspectral image under LCI and OSAVI. Different letters indicate significant different at *p* < 0.05; (**G**–**I**) vegetation diagram of vegetation index heatmap. 1, FY737; 2, ZYZ1510; 3, ZY50; 4, B11. T1, T2, T3, T4, T5 represented the seeding dates at 20th October, 30th October, 10th November, 20th November and 30th November, respectively. The dash area represents the period that cold-stress occurred. *, represents significant difference, *p* < 0.05.

Leaf samples were collected at the end of natural freezing (8 February). The dynamics of temperature during growing seasons and the field design are shown in Figure 1.

### 2.2. Unmanned Aerial Vehicle (UAV) Aerial Photography and Vegetation Index Measurement

UAV aerial photography was carried out simultaneously with the ground sampling. Visible and multispectral imaging was achieved using a Phantom 4 RTK and Phantom 4 Multispectral, respectively. DJI Terra v3.0.4. (DJI Inc., Shenzhen, China) were used for image stitching, and the image analysis was performed using ArcGIS v10.8.1 (Environmental Systems Research Institute, Redlands, CA, USA) and ENVI v5.6 (Exelis Visual Information Solutions Inc., Broomfield, CO, USA) software. Two vegetation indices, the linear combination index (LCI) and optimized soil-adjusted vegetation index (OSAVI), were selected to evaluate the overall vegetation growth of the community [23]. The calculation formulas are as follows:

LCI = (*R*850 − *R*710)/(*R*850 + *R*670)1/2

OSAVI = 1.16 × (*R*800 − *R*670)/(*R*800 + *R*670 + 0.16)

*R* is the spectral reflectance.

### 2.3. Chlorophyll Fluorescence Imaging and Histochemical Staining with DAB and NBT

At the sampling date (8 February), whole leaves of each material with different seeding times were sampled and placed into a pulse-modulated chlorophyll fluorometer system (Imaging-PAM; Walz, Effeltrich, Germany) after 30 min dark adaption to collect and photograph fluorescence parameters. The imaging of chlorophyll fluorescence was further measured using ImagingWin v2.56p software to calculate the value of Fv/Fm according to Hao et al. (2020) [24]. 

For histochemical staining, 2 cm × 2 cm leaf tissues (whole leaf tissues at T4 and T5 were sampled because of their small size), and the following staining, incubation, bleaching and imaging of O_2_^−^ and H_2_O_2_ were performed according to Ueda et al. (2013) and Meena et al. (2016), respectively [25,26].

### 2.4. Quantification of Osmoregulation Substances and Antioxidase Activities

Total soluble sugar, proline and MDA contents were measured by using assay kits purchased from Sangon Biotech (Shanghai, China) using the colorimetric method according to the manufacturer’s instructions, as were the activities of superoxide dismutase (SOD), peroxidase (POD), catalase (CAT) and ascorbate peroxidase (APX). Total soluble protein contents were measured using the Coomassie brilliant blue method.

### 2.5. Transcriptomic and Metabolomic Analysis

The leaves of each material at T1, T3 and T5 seeding time were sampled on 8th February with 3 biological replications. RNA extraction, library construction, sequencing and analysis were conducted according to Hao et al. (2022) [27]. |log_2_foldchange| > 1 and *p*-value < 0.05 were determined as differentially expressed genes (DEGs) using DEseq2 methods and the edgeR program [28]. The extraction of metabolites, UPLC-MS/MS, data quality assessment, annotation analysis, differential expression analysis, functional enrichment, etc., were measured according to Hao et al. (2022) [27]. |log_2_foldChange| > 1, *p*-value < 0.05, and variable importance in projection (VIP ≥ 1) were determined as differentially accumulated metabolites (DAMs).

### 2.6. Weighted Gene Co-Expression Network Analysis (WGCNA) of Transcriptome and Metabolome Data

WGCNAs of genes × metabolites were performed and were divided into different modules. The eigengene of the corresponding module represented the content of the gene or metabolite module. To calculate the correlation between the transcriptome module and metabolome module after dimensionality reduction, the data with at least one set of correlation p-values meeting the requirements of CCP < 0.05 were retained, and then, the heat map and correlation chord diagram were drawn.

The programming tools were R language, WGCNA package for dimensionality reduction analysis, Hmisc package for correlation analysis, pheatmap package for correlation graph visualization and Circlize package for chord diagram visualization. The specific operational methods referred to Zhang et al. (2013), Luo et al. (2013) and Yue et al. (2022) [29,30,31].

### 2.7. Statistical Analysis

Data analysis was performed using IBM SPSS v.22.0 statistical software. Duncan’s multiple range test (DMRT) was used to evaluate significant treatment effects at the significance level of *p* ≤ 0.05. GraphPad Prism v 8.0 and Origin v 2023 were used for paper drawing. The metabolomic and transcriptomic datasets presented in this study can be found in online repositories using the following accession numbers: MTBLS8179 and PRJNA991159, respectively.

## 3. Results

### 3.1. The Growth and Yield Were Significantly Repressed with the Delay in Seeding Date, While ZYZ1510 and FY737 Were Better According to UAV Imaging and Agronomic Parameters

**Figure 2:**
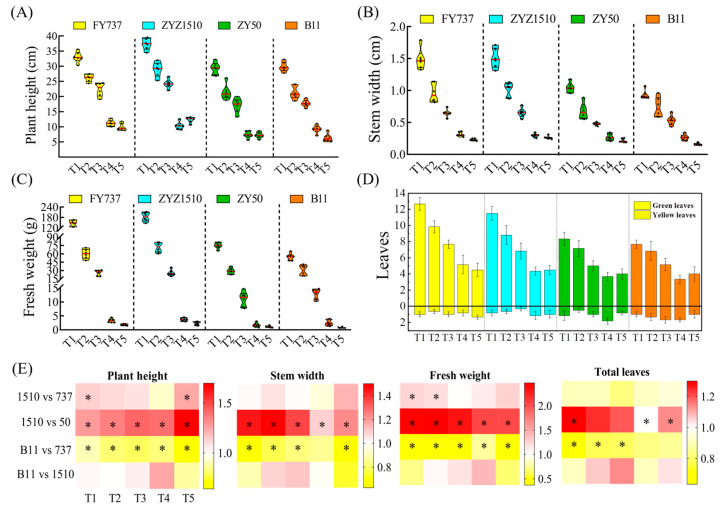
The agronomic characters of different rapeseed materials at sampling time (8th February): (**A**) plant height; (**B**) stem width; (**C**) fresh weight; (**D**) green and yellow leaf numbers. Yellow, blue, green and orange columns represent FY737. ZYZ1510, ZY50 and B11, respectively. (**E**) Heatmap representation of each agronomic character. * represents significant difference, *p* < 0.05.

According to the results of the photography and hyperspectral imaging via UAV, we can clearly observe that the growth of the rapeseed was significantly inhibited, as shown by the delay in seeding date with the drop in temperature (Figure 1A–C). FY737-T1 had the highest vegetation index, calculated by using the LCI and OSAVI methods, with values reaching 0.30 and 0.63, respectively, while ZY50-T4 had the lowest indices; the vegetation indices were 0.099 and 0.194 in LCI and OSAVI, respectively, which decreased by 67.0% and 69.2% (Figure 1B,C). However, contrary to our expectations, the vegetation index did not further decrease at T5, but slightly rebounded (Figure 1). Further measurement of agronomic traits revealed that when the seeding date was later than T3, its growth was greatly inhibited compared with earlier dates. The plant height, stem width, fresh weight and leaf numbers of the LST cultivars FY737 and ZYZ1510 were higher than those of the late-seeding-sensitive (LSS) cultivars ZY50 and B11 under all seeding dates, with the first three seeding dates being more significant (Figure 2).

For the tested LST cultivar ZYZ1510, its agronomic traits, including plant height, stem width, fresh weight and leaf numbers, were significantly higher than the LSS control ZY50 during all seeding periods (except for leaf numbers at T2 and T3). The plant height at T1 and T5, as well as the fresh weight at the T1 and T2 stages, was even higher than the LST control FY737, implying the high late seeding tolerance of ZYZ1510. For the tested LSS cultivar B11, its plant height, stem width, fresh weight and leaf numbers were obviously lower those those of the LST control FY737, except for the stem width at T4 and leaf numbers at T4 and T5 (Figure 2).

**Figure 3:**
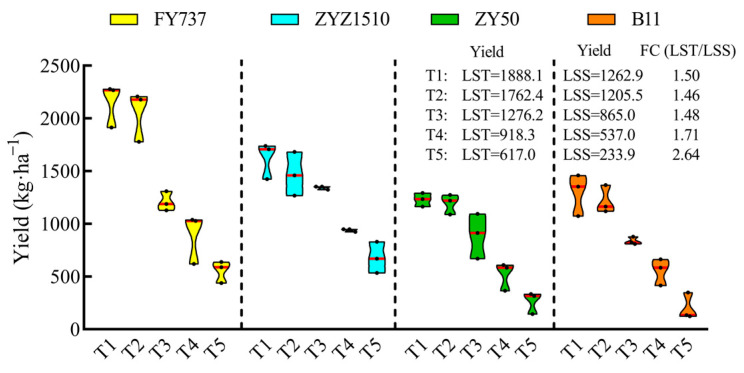
Seed yield of two late-seeding-tolerant rapeseed cultivars (FY737 and ZYZ1510) and two late-seeding-sensitive cultivars (ZY50 and B11) after manual harvest. Yields of LST and LSS represent the average yield of two LST cultivars or two LSS cultivars, respectively. FC (LST/LSS) represents the fold change in yield of LST/LSS.

The yield of LST cultivars during the entire late seeding period was higher than that of LSS. With the delay in seeding, the average yield of the LST cultivars decreased from 1888.1 kg·ha^−1^-T1 to 616.9 kg·ha^−1^-T5, while the yield of the LSS cultivars decreased from 1262.9 kg·ha^−1^-T1 to 233.9 kg·ha^−1^-T5, and the foldchange (LST/LSS) increased from 1.50 to 2.64 times (Figure 3), highlighting the advantages of LST cultivars under complex late seeding conditions.

### 3.2. Obviously Lower ROS Levels and Stress Degrees Were Detected in LST Cultivars Compared with LSS

**Figure 4:**
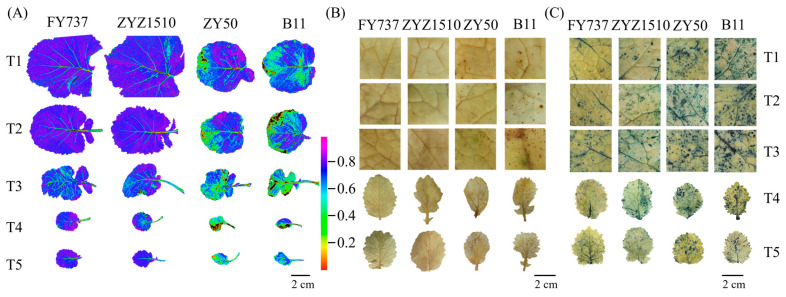
Images of different rapeseed materials with different seeding dates after undergoing natural freezing damage represented by (**A**) florescence imaging; (**B**) DAB staining; (**C**) NBT staining.

As described, the two LST cultivars showed better growth than the LSS cultivars, especially when the seeding dates were later than T3. To further determine if the growth differences were caused by stress damage, a chlorophyll florescence imaging assay was conducted, and the results showed that the lowest Fv/Fm level occurred at T3 to T4 in all cultivars, reflecting the more severe damage suffered, while both LST cultivars showed better appearances than the LSS cultivars for all seeding dates (Figure 4A).

To test if the growth differences were caused by ROS damage, we examined the accumulation levels of H_2_O_2_ and O_2_^.-^ with the DAB and NBT staining methods, respectively. Little brown spots, which represented the deposition of H_2_O_2_, were detected in both LST cultivars, except for ZYZ1510 at T5, while the areas were obviously increased in the LSS cultivars at all seeding dates, especially the dates after T3, in accordance with the florescence imaging (Figure 4B). In contrast to the DAB staining, the degrees of staining via NBT were significant, which indicated that all cultivars had suffered obvious damage from O_2_^.−^, while the LST cultivars showed a lower accumulation than the LSS cultivars for all seeding dates. Moreover, large areas of staining were detected via NBT in LSS cultivars for all seeding dates, indicating that O_2_^.-^ might be the main stressor (Figure 4C). 

### 3.3. The Stronger Activities of Antioxidase May Account for the Better Growth of LST Cultivars under Late Seeding Conditions, though Their Contents Were Decreased with the Delay in Seeding

**Figure 5:**
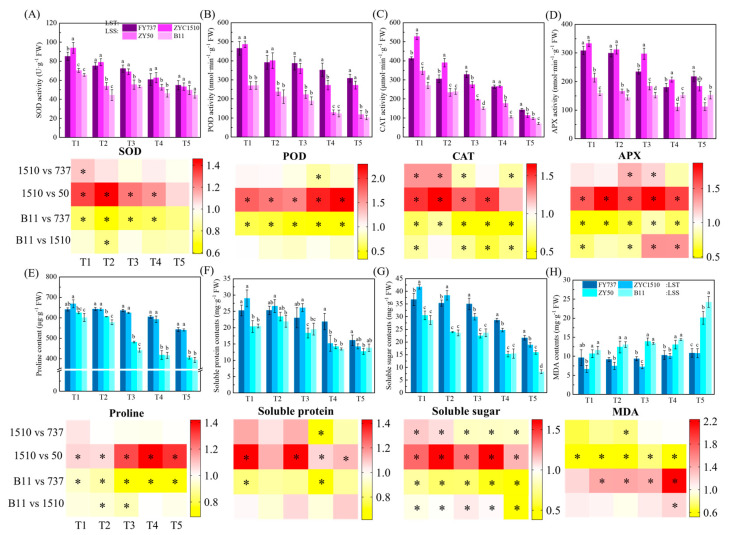
Identification of antioxidant enzyme activities, osmoregulation substance contents, MDA and their corresponding heatmap representation. (**A**–**D**) represent APX, POD, CAT and SOD activity. (**E**–**H**) represent proline, soluble protein, soluble sugar and MDA contents. LST represents late-seeding-tolerant cultivars; LSS represents late-seeding-sensitive cultivars. Different letters indicate difference at *p* < 0.05. * represents significant difference, *p* < 0.05.

In this study, the activity levels of SOD in both LST cultivars were significantly higher than those of the LSS cultivars, except for the T5 stage. The same results also appeared in POD, CAT and APX, except for the CAT and APX activity in ZYZ1510-T5 (Figure 5). This indicates that the activity levels of antioxidase in the LST cultivars were higher than those in the LSS cultivars, thus possessing higher resistance to low-temperature stress or unfavored environmental changes, enabling them to maintain stable and healthy growth under late seeding conditions. It is worth mentioning that ZYZ1510 scored higher than FY737 in terms of the activities of SOD-T1, POD-T1 and T2, APX-T3 and T4, respectively, while in the case of a delay in seeding date, this trend was reversed, such as POD in T3, T4 and T5, CAT in T3 and T5, and APX in T5, reflecting the difference in anti-stress ability in different LST cultivars and with different seeding dates.

### 3.4. The Increased Accumulation of Osmoregulation Substances Contributed to a Higher Late Seeding Tolerance

Proline contents in the LST cultivars were higher than those of the LSS cultivars except for FY737-T1, and the gap gradually increased with the delay of seeding. The average proline content in LST-T1 was 1.07 times that of LSS-T1 on average, while the fold change increased to 1.47 and 1.35 times higher in T4 and T5, respectively, indicating that LST cultivars have higher resilience in terms of proline regulation when facing unfavorable late seeding conditions, while LSS cultivars do not (Figure 5E). In terms of soluble protein, the contents in ZYZ1510 were higher than in both LSS cultivars for the first three seeding dates; however, when the seeding dates were delayed to T4 and T5, the levels became similar, but were lower than FY737 (Figure 5F). The differences were more evident in the determination of the soluble sugar content. For the first two seeding dates, ZYZ1510 showed the highest contents and had significantly higher contents than FY737 and both LSS cultivars. From T3 onwards, the content of FY737 reached the maximum, while the contents of both LST cultivars was still higher than those of the LSS cultivars, implying that soluble sugar played more important roles compared with soluble protein (Figure 5G).

As one of the indicators for identifying the degree of plant cell damage, the MDA content of both LST cultivars was significantly lower than that of the LSS cultivars, except for FY737-T1. It is worth mentioning that the fluctuation in the MDA content was relatively small in the LST cultivars throughout all seeding dates, ranging from 6.7 mg·g^−1^-T1 to 10.87 mg·g^−1^-T5 on average. However, the MDA contents were significantly increased in the LSS cultivars as the seeding date was delayed, from an average of 11.15 mg·g^−1^-T1 to 22.18 mg·g^−1^-T5, representing an increase of 98.9% (Figure 5H).

### 3.5. Identification and WGCNA Analysis of Candidate Late-Seeding-Tolerant/-Sensitive DEGs and DAMs

**Figure 6:**
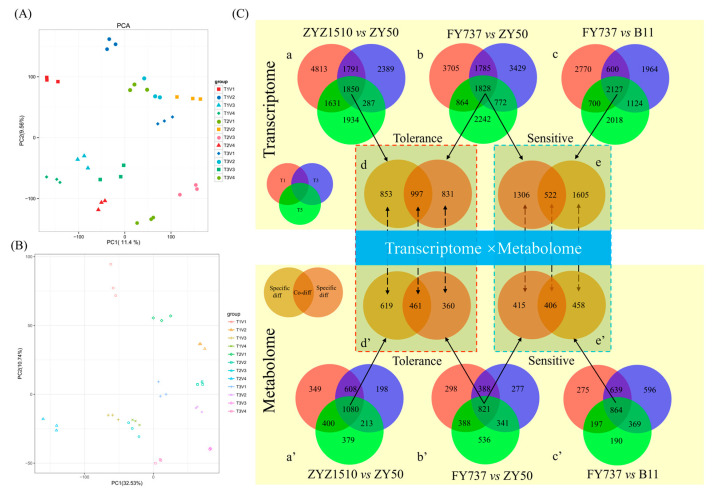
Principal component analysis (PCA) of transcriptomic and metabolomic profile of 4 rapeseed materials with 3 seeding dates, and the identification of candidate late-seeding-tolerant/-sensitive DEGs and DAMs for underlying interaction analysis. (**A**,**B**) PCA analysis of transcriptomic and metabolomic profiles; (**C**) identification of candidate late-seeding-tolerant/-sensitive DEGs and DAMs. a, b and c represent venn diagrams of DEGs at different pairwise groups. d and e represent venn diagrams of late-seeding-tolerance and late-seeding-sensitive DEGs, respectively. a’, b’, c’, d’ and e’ represent the corresponding analysis of DAMs. Red, blue and green circles represent T1, T3 and T5 seeding dates, respectively. Soil and brown circles represent the candidate late-seeding-tolerant or -sensitive DEGs or DAMs. Numbers in circles represent the count of DEGs or DAMs.

PCA analysis showed an obvious separation of transcriptome data, except for ZYZ1510-T5 and FY737-T3, indicating that different seeding dates influenced different cultivars greatly in terms of genes and metabolites (Figure 6A,B).

Furthermore, by comparing the gene expression and metabolite accumulation differences in different cultivars and for different seeding dates, we screened out 1850 DEGs and 1080 DAMs between ZYZ1510 and ZY50 from three seeding dates (Figure 6C(a,a’)), 1828 DEGs and 821 DAMs in FY737 and ZY50 (Figure 6C(b,b’)), as well as 2127 DEGs and 864 DAMs in FY737 and B11 (Figure 6C(c,c’)), which may be involved in the regulation of late seeding tolerance/sensitivity.

Taking the LSS control (ZY50) as the control and the two LST cultivars as the comparison groups, we screened out 997 co-DEGs and 461 co-DAMs that may be involved in the regulation of late seeding tolerance in both LST cultivars (Figure 6C(d,d’)). Similarly, taking the LST control (FY737) as the control and the two LSS cultivars as the comparison groups, 522 co-DEGs and 466 co-DAMs that may be involved in the regulation of late seeding sensitivity in both LSS cultivars were screened out (Figure 6C(e,e’)).

In addition, WGCNAs were used to analyze the co-expression that is regulatory of candidate late-seeding-response genes × metabolites. Genes and metabolites were divided into different modules, and the correlation between transcriptome and metabolome modules after reduced dimensionality were calculated; a heat map and correlation chord diagram were then drawn (Figure S1).

### 3.6. Candidate Genes, Metabolites and Pathways Involved in Late Seeding Response Were Further Identified via Pathway Interaction Analysis

**Figure 7:**
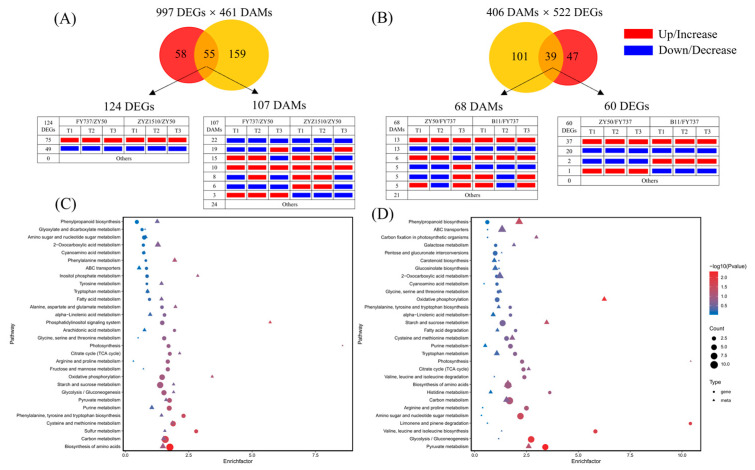
KEGG interaction analysis of candidate late-seeding-response DEGs and DAMs. (**A**) Venn diagram of pathway numbers with candidate late-seeding-tolerant DEGs and DAMs involved and the expression pattern of DEGs and DAMs involved in 55 common pathways. (**B**) Venn diagram of pathway numbers with candidate late-seeding-sensitive DEGs and DAMs involved and the expression pattern of DEGs and DAMs involved in 39 common pathways. (**C**,**D**) represent pathway enrichment analysis bubble diagrams of the top 30 pathways that might have participated in late seeding response process. Red and yellow circles represent DEGs and DAMs, respectively. Numbers in circles-crossing area represent the count of KEGG pathways in which both DEGs and DAMs are involved. Red and blue rectangles represent up/increase and down/decrease, respectively.

KEGG interaction analysis results showed that 997 co-DEGs and 461 co-DAMs that may participate in the formation of late seeding tolerance jointly participated in 55 metabolic pathways, in which 124 DEGs and 107 DAMs were involved. Among them, 75 DEGs and 10 DAMs were upregulated/increased in LST cultivars compared with ZY50, while 49 DEGs and 22 DAMs were downregulated/decreased at each seeding period (Figure 7A,B).

KEGG pathway enrichment showed that the top six metabolic pathways with the highest numbers of DEGs enriched were the biosynthesis of amino acids, carbon metabolism, starch and sucrose metabolism, oxidative phosphorylation, cysteine and methionine metabolism and glycolysis/gluconeogenesis, and purine metabolism; the number of enriched DEGs were 22, 21, 16, 12, 11 and 10, respectively. Meanwhile, pyruvate metabolism, arginine and proline metabolism, citrate cycle (TCA), photosynthesis and phenylpropanoid biosynthesis also showed DEG enrichment; the number of enriched DEGs was eight, six, five, five and five, respectively. In terms of DAMs, the top six pathways with the highest number of DAMs enriched were 2-oxocarboxylic acid metabolism, biosynthesis of amino acids, carbon metabolism, monobactam biosynthesis, purine metabolism and phenylpropanoid biosynthesis; the number of enriched DAMs was nine, nine, six, six, five and five, respectively. The pyruvate metabolism, citrate cycle (TCA), starch and sucrose metabolism, and glycolysis also showed DAM enrichment; the number of enriched DAMs was three, two, two and two, respectively (Figure 7C).

Correspondently, 522 co-DEGs and 406 co-DAMs that may participate in the formation of late seeding sensitivity jointly participated in 39 metabolic pathways, in which 60 DEGs and 68 DAMs were involved (Figure 7D). The detailed information of DEGs and DAMs that involved in 55 and 39 metabolic pathways were shown in Tables S1–S5.

### 3.7. TCA and Phenylpropanoid Biosynthesis Dominated the Regulation Network Underlying the Regulation of Late Seeding Tolerance/Sensitivity

**Figure 8:**
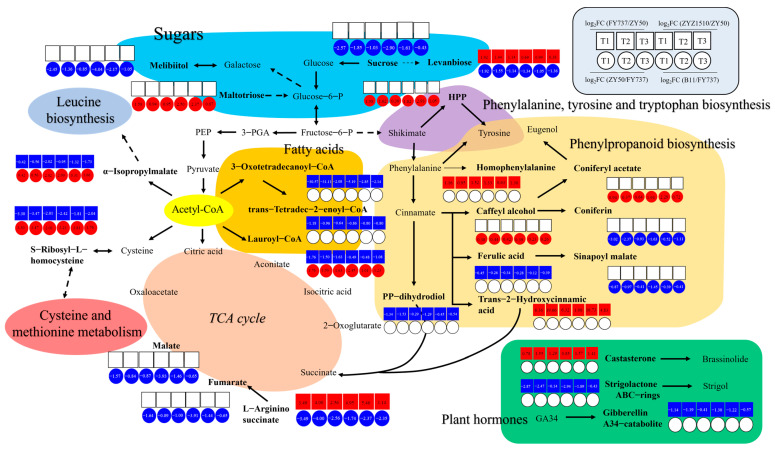
A hypothetical working model of late seeding response regulation mechanism in *Brassica napus*. Different colors represent different pathways as shown in each area.

By sorting the metabolites with the same expression trends (increased/decreased in all groups) that were determined as key DAMs involved in late seeding response regulation from the 55 and 39 metabolic pathways, we doped out a possible late seeding response regulatory network involving sugars, phenylalanine tyrosine and tryptophan biosynthesis, phenylpropanoid biosynthesis, TCA cycle, fat acid metabolism and amino acid metabolism (Figure 8).

According to the network, levanbiose and L-arginino-succinate, which are involved in sugar metabolism and the TCA cycle, respectively, are both candidate late-seeding-tolerant or -sensitive DAMs. They were upregulated in the LST cultivars and downregulated in the LSS cultivars, suggesting that they might positively regulate late seeding tolerance, while the expression patterns of α-isopropylmalate, S-ribosyl-L-homocysteine and Lauroyl-CoA were exactly the opposite, suggesting their negative effects. There were a total of eight metabolites that were upregulated in the LST cultivars and unchanged or downregulated in the LSS cultivars as positive DAMs, namely, sucrose and melibiitol in sugar metabolism; homophenylalanine, trans-2-hydroxycinnamic acid, coniferon and sinapoyl malate in phenylpropanoid biosynthesis; and malate and fumarate in the TCA cycle, indicating that an increase in their content may enhance the tolerance of late seeding, or that their decrease may make rapeseed more sensitive. Eight metabolites were downregulated in the LST cultivars and unchanged or upregulated in the LSS cultivars as negative DAMs, namely, maltotriose, HPP, coniferyl acetate, caffeyl alcohol, ferulic acid, 3-oxotetradecanoyl-CoA, trans-tetradec-2-enoyl-CoA and pp-dihydrodiol. Furthermore, three kinds of plant hormones were also identified, including one positive DAM, namely, castasterone, which is involved in brassinolide synthesis, and two negative DAMs, strigolactone ABC rings and gibberellin A34 catalite. 

### 3.8. Complicated Gene Regulation Network of Late Seeding Response under Late Seeding Conditions

Based on the six metabolic pathways involved in the regulatory network, the relevant DAMs and DEGs were selected, and the correlation network diagrams of genes × metabolites were drawn, in which metabolites are represented by red circles and genes are represented by blue squares. The correlation is represented by lines; the darker the color, the greater the correlation coefficient. Meanwhile, the log_2_foldchange of each related gene is attached. There were 31 DEGs involved in the tolerance group, while only 8 DEGs were involved in the sensitive group (Figure S2).

## 4. Discussion

The Yangtze River Basin, as an important production area of rice and rapeseed, is experiencing the problem of rice–rape stubble contradiction, which is becoming more serious as the global environment changes [32]. With a delay in its seeding date, rapeseed becomes more susceptible to the influence of extreme weather. Several researchers have studied the influence of cold and drought stress on rapeseed seedlings; however, a single stress cannot represent the complex field environment [19,20]. Furthermore, the tolerance during germination or seedling stages may not be maintained during wintering, and the merit of late-seeding-tolerant rapeseed genotypes in facing delayed field environments remains unclear [21]. Therefore, we studied the wintering ability of rapeseeds and identified that the dynamic changes in physiology, conserved genes and metabolites at different developmental stages when wintering are essential in facing delayed unfavorable growth environment conditions.

### 4.1. Rapeseed Production Was Largely Restricted by Climate Change under the Rice–Rape Rotation System, While LST Cultivars Showed Better Yield Resilience than LSS Cultivars

Abiotic and biotic factors generated by climate change greatly affect global agriculture production and food security, which will reshape crops through processes of phenotypic adjustment, genetic adaptation and epigenetic modifications [33,34]. Analyses of crop adaptive actions to climate change focusing on physiological, metabolic, developmental and phenological mechanisms have shown them to play a vital part in the acclimation to climate change [35,36]. However, restricted by limited knowledge and characterization, single-laboratory or potted research cannot fully simulate the changed environment and the response of plants to climate change [37]. Taking the rice–rape rotation system in the Yangtze River Basin as an example, the seeding time of rapeseeds has been largely postponed with the delay in the rice harvest. Meanwhile, with the more frequent occurrence of extreme weather resulting from global climate change, the requirements of rapeseed’s stress resistance have been greatly raised, especially when wintering. According to reports, cold stress includes chilling (0–15 °C) and freezing stress (<0 °C) [38], and the rapeseed in our study encountered an extremely long chilling stress period from 28th November, 2022, to 8th February, 2023 (Figure 1D). The seed germination and seedling emergence stages of rapeseed are the most sensitive to abiotic or biotic stress, which directly determines a rapeseed population’s density and grain yield [39]. Using joint analysis with the temperature data, we speculated that the appropriate temperature led to the rapeseed growing well in T1 and T2; however, when we came to T4, the seedlings that were less than 10 days old were not vigorous enough to resist the following sudden drop in temperature and drastic cold stress on the 29th November, hence resulting in the formation of a peak–valley cycle at T4 (Figure 1). As for T5, the seeds delayed germination when facing harsh environmental conditions until the weather became appropriate, and hence, they showed a slight rebound in growth appearance compared with T4 (Figure 1). In general, however, the LST cultivars showed a higher yield resilience compared with the LSS cultivars; with the delay in seeding dates, the fold change (LST/LSS) of the yield even increased from 1.50 to 2.64 times in the LST cultivars compared with the LSS cultivars, and the yield of LST-T5 was even higher than that of LSS-T4, implying that traditional rapeseed cultivars facie partial or total crop failures with the delay in seeding dates, while new adapted cultivars can still gain a respectable yield (Figure 3).

### 4.2. Antioxidase and Osmoregulation Substances Played Important Roles in the Adaption Process of Rapeseed to Late Seeding

Under stress, the plant homeostatic apparatus was amended by generating an increased reactive oxygen species (ROS) in plant cells, which were harmful to plant development, although ROS are considered major signaling molecules regulating abiotic and biotic stress responses [40]. Research has shown that high-chilling-tolerance cultivars accumulated more antioxidants, enzymes and osmoregulation substances in the anti-stress and environment adaption process compared with low-chilling-tolerance cultivars [41,42]. In more detail, the functions of the soluble protein and carbohydrate levels on the improvement in freezing tolerance were validated in sauvignon [43], while the functions of increased proline and carbohydrates under cold stress were also verified in alfalfa [44]. Furthermore, the enhanced chilling tolerance in watermelon achieved via suboptimal temperature acclimation was contributed to by improved photosynthetic adaptability and osmoregulation ability from substances such as proline, soluble sugar and sucrose [45]. From the conclusions above, we determined that the functions of osmoregulation substances and antioxidases were critical to stress; however, in this study, we found that as seeding dates were delayed, osmoregulation substances consistent with antioxidases decreased, which might contribute to the weakening of the environmental adaptability. However, both LST cultivars demonstrated better levels of POD, proline and soluble sugar contents among almost all seeding dates, which may explain their better growth and high late seeding tolerance.

### 4.3. Interaction Analysis of Multi-Omics Contributed to the Mechanism Identification of Late Seeding Tolerance

With the metabolomics and transcriptomics data from the LST and LSS cultivars at three seeding dates, we were able to obtain a global and comprehensive view of the dynamic changes of metabolites, genes and their interactions involved in the resistance to gradually delayed seeding conditions. Through differentially expressed/accumulated analysis and KEGG pathway interaction analysis, we were able to dope out a possible late seeding response regulatory network as shown in Figure 8. As reported, the TCA cycle, as the ultimate metabolic pathway and also the hub of the three major nutrients (sugars, lipids and amino acids), is critical in many developmental processes for plant growth, such as cotyledon development, flower development and so on [46,47]. The phenylpropanoid biosynthetic pathway, as the most important secondary metabolism pathway, regulates the biosynthesis of numerous secondary metabolites in plants, such as flavonoids, flavones, lignin, coumarin, anthocyanin and so on, and is crucial in plants’ growth, development and response to adversity [48,49,50]. In more detail, isocitrate dehydrogenase (*ICDH*) is a TCA cycle enzyme; Lee et al. (2009) found that the activity of NADP-specific isocitrate dehydrogenase (*NADP-ICDH*) was induced via chilling stress in rice roots [51]. In this study, we found that *NADP-ICDH* (*BnaA03G0059700ZS*) was significantly higher in LST cultivars, indicating its positive effects on late seeding (Figure S2). Pyruvate kinase (*PK*) is one of the limiting enzymes of glycolysis and catalyzes the synthesis of pyruvic acid. Yu et al. (2022) found that the transcriptional level of *PK* in *Brassica napus* was significantly increased in response to freezing stress. The function of *PK* in cold stress has also been confirmed in wheat [52]. Zhang et al. (2023) found that the regulation of *PK* in terms of cold tolerance of wheat and the exogenous application of pyruvic acid can significantly enhance the tolerance of wheat [53]. In this study, we found that pyruvate kinase (*BnaA03G0034600ZS*) was significantly upregulated in the LST cultivars compared with the LSS cultivars, demonstrating its important role in improving late seeding tolerance (Figure S2). 

Phosphoenolpyruvate carboxykinase, ubiquitous in flowering plants, was isolated and cloned as a new cold-induced gene and named *BnPEPCK* by Sáez-Vásquez et al. (1995); however, the gene functions related to cold tolerance still require further analysis [54]. In this study, we found that *BnPEPCK* (*BnaA06G0306000ZS*) was upregulated in the LSS cultivars, suggesting that it may negatively regulate late seeding tolerance, but its function still requires further validation (Figure S2). Furthermore, newgene_7532, encoding an uncharacterized protein, LOC10631242, was significantly upregulated in the LSS cultivars, with an average upregulation range of 8.88 < log_2_foldchange < 10.31. It was also significantly downregulated in LST cultivars, indicating its negative role in the resistance to late seeding (Figure S2).

Taken together with the study of gene expression via transcriptome data and the correlation network analysis of genes × metabolites, we finally screened out 31 + 8 DEGs involved in late seeding response regulation, including *ICDH*, *PK* and *PEPCK*, which were demonstrated to participate in environmental adaption regulation, as was newgene_7532. Therefore, we speculated that the regulators of the TCA-dominated primary metabolism, such as sugars and amino acid, and the phenylpropanoid-dominated secondary metabolism might be the keys to rapeseed’s response to late seeding conditions or an unfavorable change in environment. 

## 5. Conclusions

This study demonstrated the adaption process of rapeseed in the YRB under unfavorable late seeding conditions and validated the late-seeding-tolerant *Brassica napus* genotype ZYZ1510 and the late-seeding-sensitive genotype B11. By focusing on agronomic characters, antioxidant systems and osmoregulation substances, our findings will provide a great reference for future directions of rapeseed breeding under late seeding conditions. Furthermore, throughout the interaction analysis of genes × metabolites, we have successfully identified candidate late-seeding-response genes and metabolites and proposed a probable regulation pathway of rapeseed in response to late seeding environments, which has significant implications for future genetic improvement of rapeseed and can contribute to future oil security and sustainability.

## Data Availability

Data will be made available on request.

## Supplementary Materials

The following supporting information can be downloaded at: https://www.mdpi.com/article/10.3390/antiox12111915/s1. Table S1: Detail of the expression model of genes and metabolites involved in late-seeding-response; Table S2: Details of the candidate DEGs involved in the 39 late-seeding-sensitive pathways; Table S3: Details of the candidate DAMs involved in the 39 late-seeding-sensitive pathways; Table S4: Details of the candidate DEGs involved in the 55 late-seeding-tolerance pathways; Table S5: Details of the candidate DAMs involved in the 55 late-seeding-tolerance pathways; Figure S1: WGCNA analysis of genes × metabolites; Figure S2: Correlation network diagrams of genes × metabolites.
