# Supplementary material for: How Antioxidants, Osmoregulation, Genes and Metabolites Regulate the Late Seeding Tolerance of Rapeseeds (Brassica napus L.) during Wintering
Source: Antioxidants (Basel). 2023 Oct 26;12(11):1915. doi: 10.3390/antiox12111915 (PMC10669261; doi:10.3390/antiox12111915)
Supplement: Supplementary file 1 [file antioxidants-12-01915-s001.zip › Supplementary figures.pptx]

## Slide 1
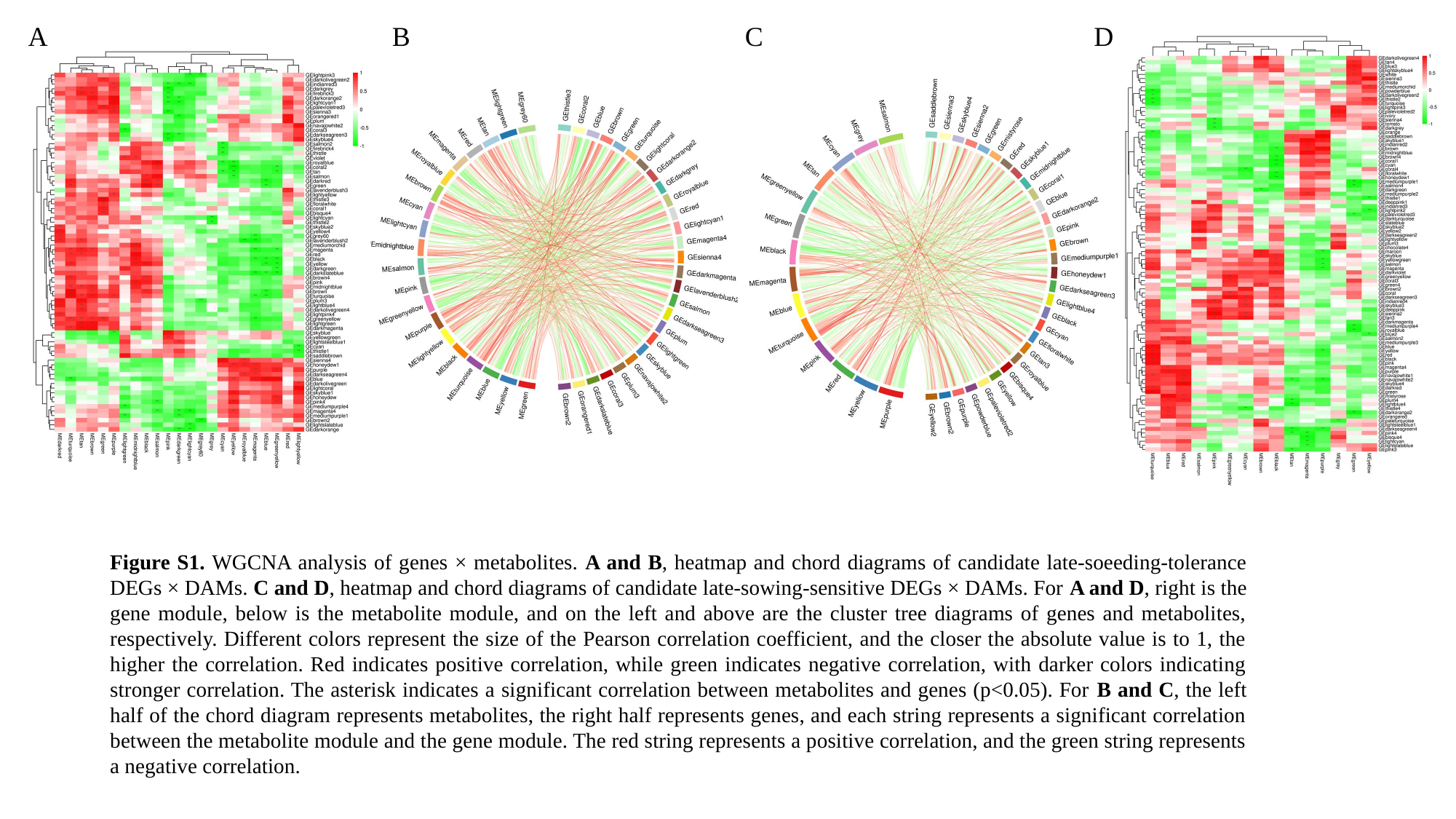

A
B
C
D
Figure S1. WGCNA analysis of genes × metabolites. A and B, heatmap and chord diagrams of candidate late-soeeding-tolerance DEGs × DAMs. C and D, heatmap and chord diagrams of candidate late-sowing-sensitive DEGs × DAMs. For A and D, right is the gene module, below is the metabolite module, and on the left and above are the cluster tree diagrams of genes and metabolites, respectively. Different colors represent the size of the Pearson correlation coefficient, and the closer the absolute value is to 1, the higher the correlation. Red indicates positive correlation, while green indicates negative correlation, with darker colors indicating stronger correlation. The asterisk indicates a significant correlation between metabolites and genes (p<0.05). For B and C, the left half of the chord diagram represents metabolites, the right half represents genes, and each string represents a significant correlation between the metabolite module and the gene module. The red string represents a positive correlation, and the green string represents a negative correlation.

## Slide 2
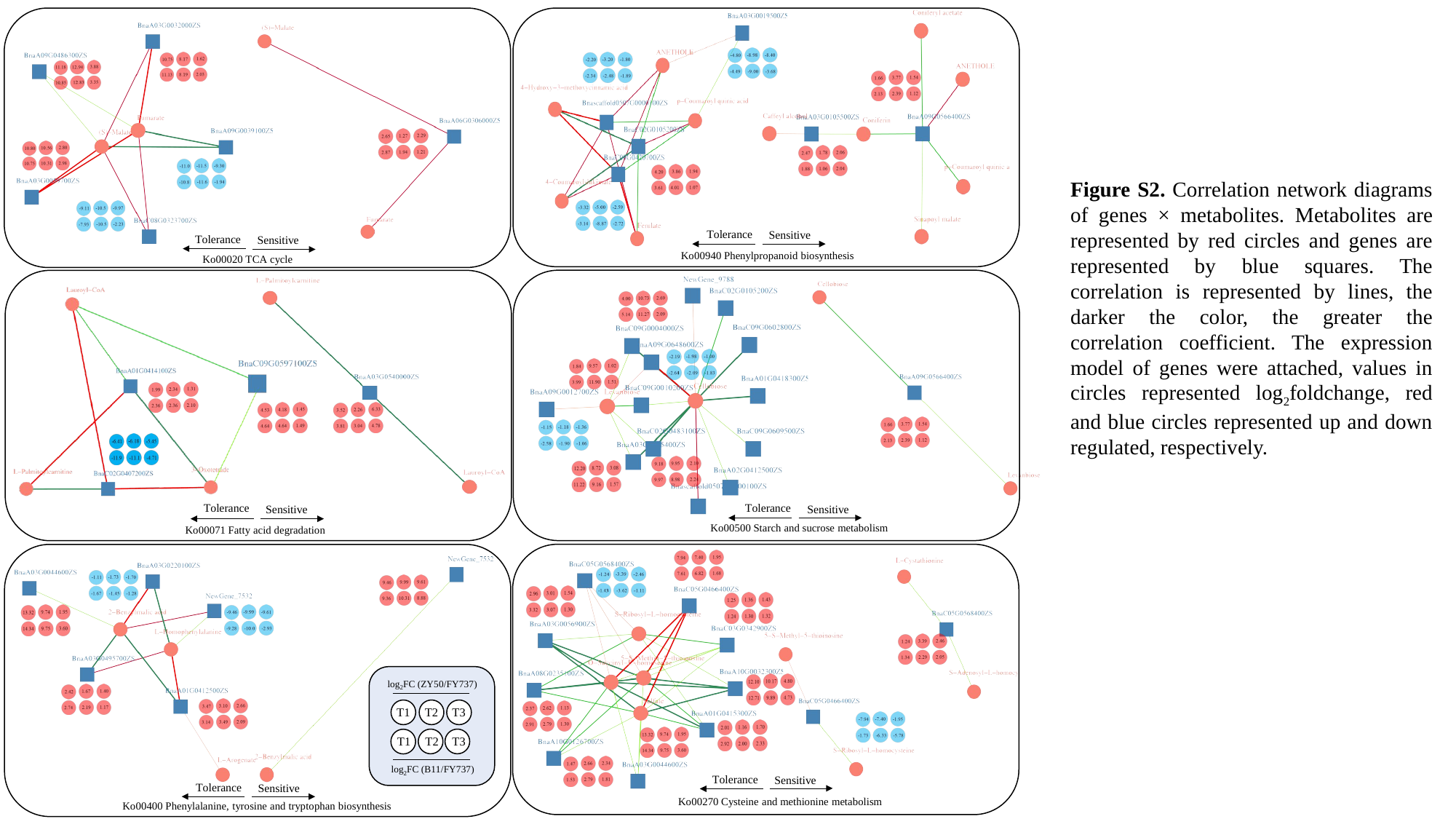

Figure S2. Correlation network diagrams of genes × metabolites. Metabolites are represented by red circles and genes are represented by blue squares. The correlation is represented by lines, the darker the color, the greater the correlation coefficient. The expression model of genes were attached, values in circles represented log2foldchange, red and blue circles represented up and down regulated, respectively.
